# Testicular cancer among Saudi adults: Hands on a nationwide Cancer Registry over 10 years

**DOI:** 10.1080/2090598X.2022.2084902

**Published:** 2022-06-10

**Authors:** Yasser A. Noureldin, Muhannad Q. Alqirnas, Meshal F. Aljarallah, Omar B Alfraidi, Mohammad A. Alghafees, Yahia Ghazwani, Abdullah Alkhayal

**Affiliations:** a Division of Urology, King Abdulaziz Medical City, Riyadh, Saudi Arabia; b King Abdullah International Medical Research Center, Riyadh, Saudi Arabia; c Department of Urology, Faculty of Medicine, Benha University, Benha, Egypt; d College of Medicine, King Saud Bin Abdulaziz University for Health Sciences, Riyadh, Saudi Arabia

**Keywords:** Testicular cancer, tumors, adults, Saudi Arabia

## Abstract

**Background/objective:**

Testicular cancer (TC) is one of the most curable solid malignancies affecting young adults. The objective of this study was to identify factors affecting survival of Saudi adults who were diagnosed with testicular cancer over 10 years.

**Methods:**

This was a retrospective study with data extracted from the Saudi Cancer Registry for Saudi Adults diagnosed with TC from 2008 to 2017. We collected demographic information, including age, marital status, region of residency, year of diagnosis, and the survival status. In addition, the tumor factors included the basis of diagnosis, the origin of the tumor, histopathological group and subtype, and tumor behavior, stage, and laterality were collected.

**Results:**

A total of 869 patients were included, with a median age of 30 (**IQR**: 25–38). The highest percentage of the cases was 37.5% (326) in the Central region, followed by the Western region 24.6% (214). The primary site of the tumor was the testis 96.9% (842), 3.1% (27) in the undescended testis. The histopathological examination revealed seminoma in 44.8% (389), 33.5% (291) mixed germ cell tumor, 8.4% (73) embryonal carcinoma, 6.1% (53) teratoma, 2.6% (23) yolk sac tumor, 1.6% (14) choriocarcinoma, 0.3% (3) Leydig cell tumor, and 2.6% (23) sarcomas. Kaplan-Meier analysis revealed significant association between survival and the age groups (p = 0.001), histopathology group (p 0.04), histopathology subtypes (p = 0.01), and the stage of the tumor (p < 0.001).

**Conclusions:**

A notable increase in the incidence of TC among Saudi adults was seen, with a mortality rate of 5.4% over a period of 10 years. Longer survival was associated with age groups, seminomatous germ cell tumor, and lower tumor stage.

## Introduction

While testicular germ cell tumors (T-GCTs) make up only 0.5% of pediatric malignancies and less than 2% of adult malignancies, they comprise 14% of adolescent malignancies, making it the most common solid tumor in this age group [1]. Furthermore, the incidence of TC is increasing globally [2]. The overall 5-year survival rate of TC is ~95%, and the 15-year survival rate for patients diagnosed with stage I disease is ~99% [3]. TC is classified histologically as germ-cell tumors, which account for 95% of the cases and non-germ cell tumors account for the remaining 5% [4]. A major risk factor that increases the chance of developing TC by nearly five times is cryptorchidism, or undescended testis [5]. As cryptorchidism is more common on the right side, a slight increase in incidence in right-sided testicular cancer is observed [6]. Also, the self-history of TC increases the risk by 5% in the contralateral testis [7]. From 1994 to 2013, 1004 cases of testicular cancer among adult Saudis were reported to the Saudi Cancer Registry **(SCR**), with a steadily significant increase in incidence rate reaching an annual rate of 94 cases in 2013 [8]. Unfortunately, the survival rate of TC in Saudi Arabia has been poorly studied. Therefore, this study aimed to investigate the incidence, mortality, and survival rate of TC in Saudi Arabia over a period of 10 years. In addition, we aimed to identify factors affecting survival of Saudi adults who were diagnosed with testicular cancer over 10 years period.

### Patients and methods

This retrospective study involved all adult patients diagnosed with a primary testicular tumor, 18 years and older, from 1 January 2008 to 31 December 2017. The exclusion criteria were non-Saudis, patients younger than 18 years, and tumors of a secondary origin. Ethical approval (NRC21.223.05) was obtained from King Abdullah International Medical Research Center (KAIMRC) prior to the beginning of this study.

### Data collection

The Saudi Cancer Registry under the Saudi Health Council (SHC) provided the required data for this study, which included tumor data from hospitals across the Kingdom of Saudi Arabia, including hospitals from the private sector, Ministry of Health, and military hospitals. The data from the five regional offices of the SHC are sent to the main office in Riyadh which is responsible for data analysis and periodic reports. All the eligible patients were included. We collected tumor factors including the basis of diagnosis, the origin of the tumor, histopathological group, histopathological subtype, tumor behavior, stage of the tumor, and laterality. In addition, demographic variables such as age, marital status, region of residency, year of diagnosis, and the survival status over 10 years were collected. Stages of tumor are defined as, stage 1: tumor limited to testis with no nodal or extra nodal involvement, stage 2: metastasis of the testicular tumor to the retroperotoneal lymph nodes, stage 3: distant metastasis of the testicular cancer to a non-regional nodal or extranodal tissue involvement [9].

### Data analysis

The data analysis was performed with the Statistical Package for the Social Sciences, SPSS 26th version from IBM. Frequency and percentages were used to display the categorical variables, and the median and interquartile range (IQR) for the continuous variables. A Kaplan–Meier survival analysis was generated to determine the variables affecting survival and to construct survival curves for the different variables (age groups, histopathology groups, the histopathology subtypes, and stage of tumor). The p-values of Kaplan-Meier Analyses were generated using the log-rank test. The level of significance was set at p < 0.05.

## Results

A total of 869 patients were included in this study with a median age of 30 (**IQR**: 25–38). The socio-demographic profile of the patients was described in Table 1. The majority 24.9% (216) were in the 25–29 year age group, 4.3% (37) in the 18–19 year age group, 19.3% (168) in the 20–24 year age group, and 18% (156) in the 30–34 year age group (Table 1). In terms of marital status, 45.3% (394) were single, 54.2% (471) married, 0.5% (4) divorced. As for the place of residency, 37.5% (326) were living in the Central region (Table 1).

**Table 1.** Socio-Demographic profile of the participants (n = 869).

**Age Groups (yr)**		
18–19	37	4.3
20–24	168	19.3
25–29	216	24.9
30–34	156	18
35–39	104	12
40–44	66	7.6
45–49	52	6
50–54	33	3.8
55–59	7	0.8
60–64	7	0.8
65–69	5	0.6
70+	18	2.1
**Marital Status**		
Single	394	45.3
Married	471	54.2
Divorced	4	0.5
**Place of Residency**		
Central Region	326	37.5
Eastern Region	184	21.2
Northern Region	56	6.4
Western Region	214	24.6
Southern Region	89	10.2

In terms of tumor profile, the primary origin of the tumor was in a normally descended testis in the majority of cases 842 (96.9%) (Table 2). For the tumor Histopathology group, 389 (44.8%) of patients had seminoma, 454 (52.2%) had nonseminomatous germ cell tumors (NSGCT), and 26 (3%) had non-germ cell tumors. Nonseminomatous subtype and non germ-cell tumor subtypes were described in Table 2. The majority were stage 1 639 (73.5%) and right side tumor 475 (54.7%). The behavior of the tumor was malignancy in all the patients. The basis for the diagnosis was clinical examination, imaging, and histopathology.

**Table 2.** Tumour profile.

Tumor Profile (n = 869)	n	%
**Primary Site**		
**Descended Testis**	842	96.9
**Undescended testis**	27	3.1
**Histopathology Subtype**		
Seminoma	389	44.8
Mixed germ cell tumor	291	33.5
Embryonal carcinoma	73	8.4
Teratoma	53	6.1
Yolk sac tumor	23	2.6
Testicular choriocarcinoma	14	1.6
Leydig cell tumors	3	0.3
Sarcoma	23	2.6
**Stage**		
Stage I	639	73.5
Stage II	71	8.2
Stage III	159	18.3
**Lateralization**		
Right	475	54.7
Left	368	42.3
Bilateral	26	3
**Year of Diagnosis**		
2008	66	7.59
2009	69	7.94
2010	70	8
2011	78	8.97
2012	81	9.32
2013	89	10.24
2014	96	11.04
2015	99	11.39
2016	106	12.19
2017	115	13.23

**Figure 1.**
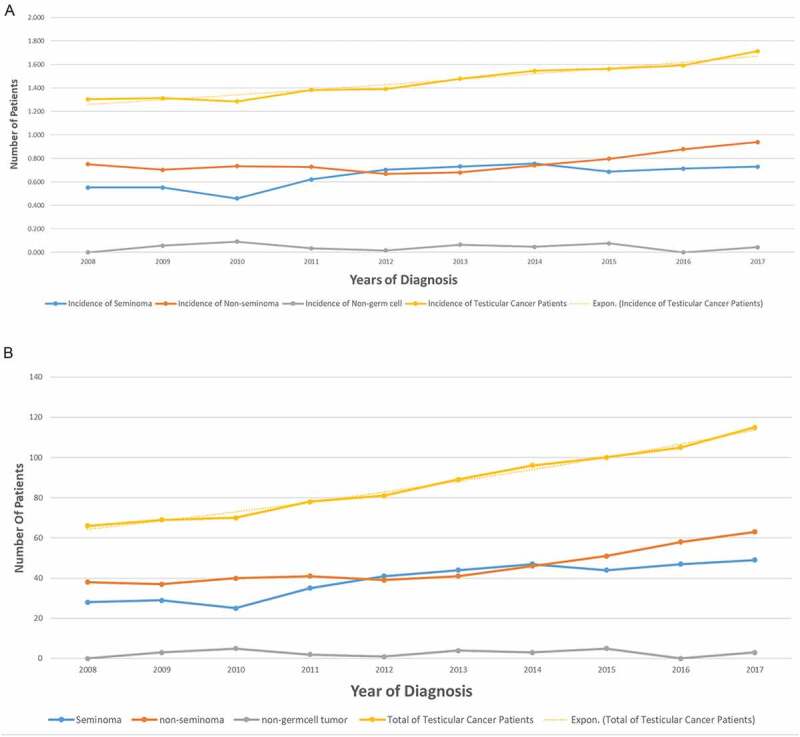
(a) Incidence of testicular cancer In Saudi Arabia over the years per 100,000. (b) Incidence of testicular cancer in Saudi Arabia over the years.

For the year of incidence and prevalence of TC per 100,000 across the years were described in Figure 1(a,b). The overall trend of TC incidence showed a slow but steady increase over the years. The highest incidence of overall TC (1.714) was observed in 2017, with the lowest incidence (1.256) observed in 2010 (Figure 1(a,b)). A notable increase in the incidence of TC was seen in all histopathological groups and subtypes over years. The overall incidence of TC has gradually increased from 1.256 to 1.714 **(per 100,000)** at a rate of 36.5% throughout 2008–2017. Non-seminoma was the most common subtype with gradual increase in incidence rate by 56.8% (2008–2017) from 38 cases to 63, while seminomas incidence rate increased by 75% (2008–2017) from 28 cases to 49. Non-germ cell tumors showed a flat curve with cases ranging from 0 to 5 per year (Figure 1(b)).

**Figure 2.**
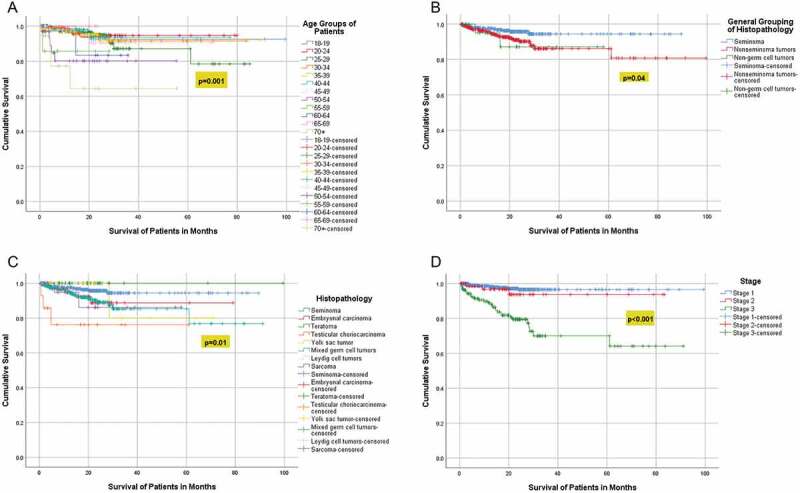
(a) Survival analysis according to the age groups (b) Survival analysis according to the Histopathology Groups. (c) Survival analysis according to the histopathological subtypes (d) Survival analysis according to the tumor stage.

**Figure 3.**
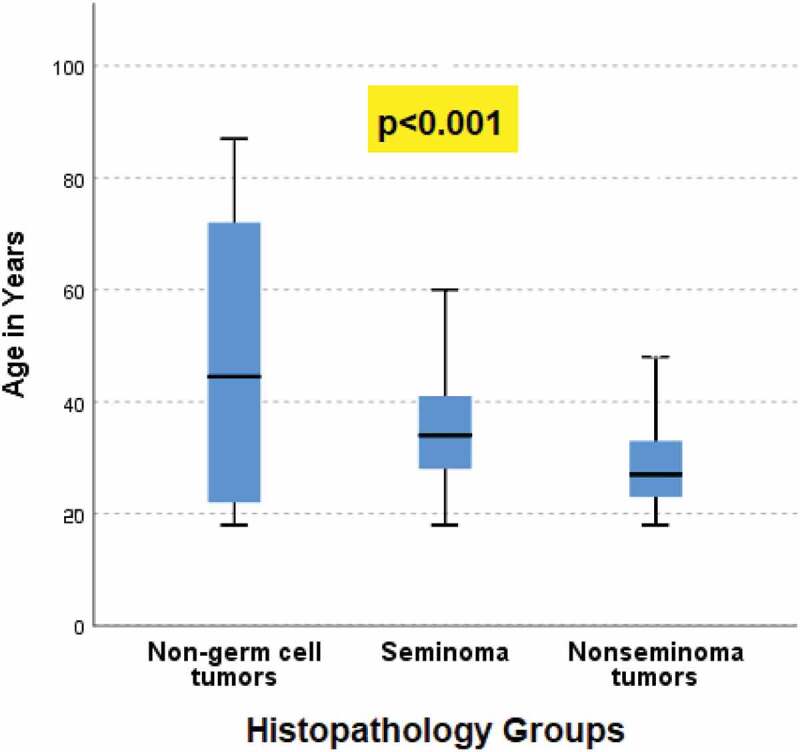
Association between histopathology groups (non-germ cell tumors, seminoma, nonseminoma tumors) and age (in years).

**Figure 4.**
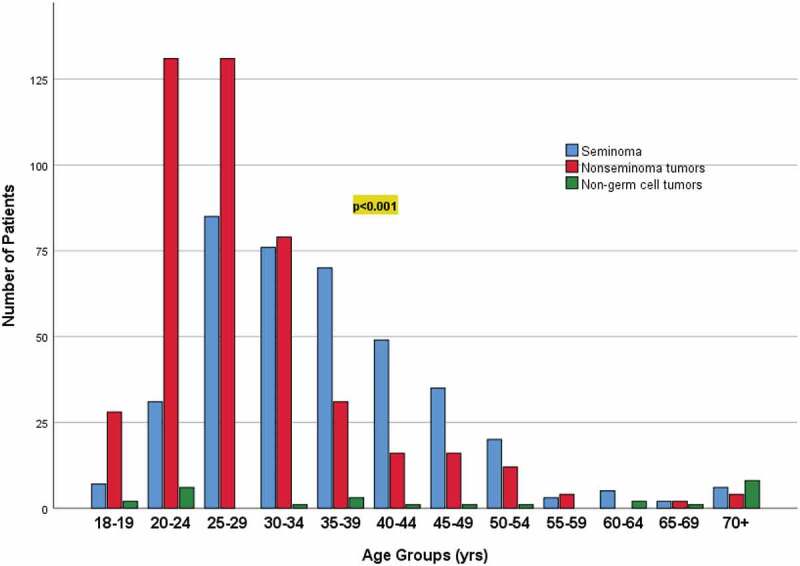
Association between histopathology groups (seminoma, nonseminoma tumors, non-germ cell tumors) and age groups (in years).

Kaplan-Meier analysis revealed significant association between survival and the age groups (p = 0.001), histopathology group (p 0.04), histopathology subtype (p = 0.01), and the stage of the tumor (p < 0.001) (Figure 2(a–d)), (Table 3). Over the 10 years, the mortality rate was 5.4% (47) and all the patients died of a cause secondary to TC; 27.6% (12) died with stage I, 6.4% (3) died with stage II, and the majority 66% (31) died with stage III (p < 0.001), (Table 4). There was a significant association between age and histopathological groups (p < 0.001), and age groups and histopathological groups (p < 0.001) (Figures 3 and 4). For the 47 (5.4%) patients who passed away, the median time to death was 9.7 months (IQR: 3.2–16.9), and 31 of them had stage 3 TC.

**Table 3.** Kaplan–Meier survival analysis across different factors.

Factor	Estimated Survival(Months)	Standard Deviationof Error	p-value
**Histopathology Group**			**0.04**
Seminoma	85.1	1.278	
Nonseminoma	85.9	2.951	
Non-germ cell tumor	52.0	3.951	
**Stage of Tumor**			**<0.001**
Stage 1	96.4	0.860	
Stage 2	78.9	2.553	
Stage 3	66.7	4.11

**Table 4.** Deceased versus alive according to the stage of tumor.

Status	Stage 1 (n)	Stage 2 (n)	Stage 3 (n)	p-Value
Dead	12	3	31	<0.001
Alive	626	68	128	
Total	639	71	159	

## Discussion

The geographical incidence of tumors is usually influenced by environmental, racial, and cultural factors. However, in Saudi Arabia, our understanding of TC risk factors, prevalence, incidence rate, and age distribution is insufficient due to underreporting in the literature. This study aimed to fill this deficit with a decade of data and a survival analysis.

### Patient demographics

We reported that 86% of the patients with TC were 44 years old and younger. Current statistics indicate TC is largely a disease of young and middle-aged men. However, about 6% of cases occur in children and teens, and about 8% occur in men over the age of 55, with the average age at the time of diagnosis of 33 [10]. A report published by Chia et al explored the international trends in the incidence of TC reported that the most prevalent age group affected by TC was young adult men aged 15–40 years [11]. This was corroborated by Baird et al that recorded an incidence of 5.6 cases per 100,000 persons for males aged 15 to 34 years in the United States, noting that the incidence rates peaked in those aged between 30 to 34 years of age (14.6 cases per 100,000 persons) [12]. In comparison, the age of the patients from the Saudi national data on testicular cancer for over two decades ranged between 15–93 years, with a mean of 34.5 years. The most affected age group was 20–34 years, where 51% of all TC accumulated [8].

### Tumor profile

Seminomatous germ cell tumours were present in 44.8% of the study population, and 52.2% had NSGCTs (including mixed germ cell tumors, embryonal carcinomas, teratomas, yolk sac tumors, choriocarcinomas), and 3% had non-germ cell tumors including Leydig cell tumors and sarcomas. These findings are slightly lower than what reported in the literature. For instance, Dieckmann et al reported that 60.2% of their 422 patient cohort presented with seminomas, with 39.8% NSGCTs. The same study reported that the primary tumor in patients was primarily located on the right side (47.6%), followed by the left (47.4%), and 3.5% bilateral [13]. This was comparable to our findings as the right side incidence was more prevalent. Moreover, a study by Abomelha reported on the characteristics of TC in Saudi Arabia over 20 years, with the findings substantiating the tumor profile described in our study [8].

### Incidence and mortality rate of TC

Our study demonstrated a gradual increase in the incidence of TC over the years, with the number of cases peaking at an incidence of 1.71 per 100,000 in 2017 with a mortality rate of 5.4% over the 10-year period (2008–2017). The study conducted by Qiliang Cai and colleagues analyzed existing data on TC morbidity and mortality from 1990 to 2016 showed a significant upward trend in the incidence and a slow upward trend in the mortality of TC from 1990 to 2016, and predicted the increase of age-standardized incidence rate and the downward trend of age-standardized death rate in 2017 to 2030 [14]. Furthermore, Shanmugalingam and colleagues observed the same trend across Europe, with the annual percentage changes for the incidence of TC increasing significantly in Sweden (2.4%), United Kingdom (2.9%), and Spain (5.0%), as well as Australia (3.0%) and China (3.5%) [15]. Identifying the major causes for the increased global incidence remains a challenge. However, this could be attributed to the attenuation of TC risk in younger generations [16], resulting in an increase in TC reporting. Despite the notable increase in TC incidence in this study and similar studies, a significant downward trend has been observed for the mortality rates [15]. This is most likely the result of the enhanced therapeutics available for the treatment of TC.

### Factors associated with mortality and survival

Testicular cancer incidence is strongly related to age, with the highest incidence rates being in the 30 to 34 age group [17]. However, old age is significantly associated with lower survival following a diagnosis of TC. A study by Verhoeven and colleagues observed a significant decrease in the overall five-year survival in patients over 60 years when compared to those in younger age categories in the Netherlands [18]. Despite no current literature discussing these findings, several studies suggested that the disparities in access to healthcare in rural areas significantly reduce the overall survival of patients with TC [18]. Other factors that influence the testicular cancer-specific survival are age at diagnosis, socio-economic status, race, marital status, tumour stage, radiotherapy, and retroperitoneal lymph node dissection [19,20].

Rural regions historically have a greater overall cancer incidence than urban regions, although some key differences exist regarding the site. There was a 2.6% increased rate of TC in U.S. urban as compared with rural geographic regions between 2011 and 2015. When the geographic region is disaggregated, rural regions see higher rates than urban [21]. Our study also noted that seminomas and early stage TC had significantly better survival rate as corroborated by a study done by Stang and colleagues which reported lower five-year relative survival for NSGCT (93.3%) compared to seminomas (97.6%) in a cohort of 22,282 patients [22]. No significant difference between marital status and survival was observed in our cohort. Although the relationship between marital status and the survival of patients with TC is unclear, marital status was an independent predictor of improved overall and cancer-specific survival in men with testis cancer [23].

### Limitations

One limitation in our study is the underreporting in cancer registries. This was highlighted in the study by He and colleagues who reported that misreporting and underreporting in cancer registries is unavoidable [24]. In addition, the study’s retrospective nature might also have introduced some bias due to the unavailability of some data. Nevertheless, this study included a large sample size of TC cases from Saudi Arabia and characterized the demographic variables and the survival over a 10-year period. Unfortunately, the SCR did not account for the ethnic background of the patients which could have implicated racial differences with TC incidence. Another limitation was the unavailability of serum tumor markers (alpha-fetoprotein, human chorionic gonadotropin, and lactate dehydrogenase), nor did it include methods of treatment which might have shown the effectiveness of the treatment modalities. However, despite the rarity of TC in Saudi Arabia, we expect this study to aid physicians and prospecting researchers to expound upon TC research as it is slowly but steadily increasing both in Saudi Arabia and globally.

## Conclusion

The results of this study corroborate the current literature regarding the demographic variables and the prevalence of TC. Over a period of 10 years, the mortality rate of testicular cancer among Saudi adults was 5.4%. Longer survival was associated with age groups, seminomatous germ cell tumor, and lower tumor stage.

## Data Availability

Data available on request from the authors

## Abbreviations

**TC**: Testicular Cancer

**CT**: Computed Tomography

**SCR**: Saudi Cancer Registry

**SHC**: Saudi Health Council

**NSGCT**: non-seminomatous germ cell tumors
